# Calcinoses scrotales

**DOI:** 10.11604/pamj.2014.18.297.4971

**Published:** 2014-08-14

**Authors:** Abdou Ahlam, Badreddine Hassam

**Affiliations:** 1 Service de Dermatologie, CHU Ibn Sina, Université Med V, Souissi, Rabat, Maroc

**Keywords:** Calcinose scrotale idiopathique, histologie, métabolisme phosphocalcique, Idiopathic scrotal calcinosis, histology, calcium and phosphate metabolism

## Image en medicine

La calcinose scrotale idiopathique est une affection indolente bénigne et rare survenant en l'absence d'anomalie du métabolisme phosphocalcique. Elle apparaît chez les hommes de 20 à 40 ans le plus souvent de peau noire, bien que des formes pédiatriques et gériatriques aient été décrites. Les nodules sont asymptomatiques uniques ou multiples pouvant mesurer jusqu’à 7 cm. La consultation est motivée par la gène esthétique qu'elle occasionne. L'histologie met en évidence la présence de dépôts calciques basophiles (coloration VonKossa) dans le derme scrotal et les fibromes calcifiés, entourés de granulomes à cellules géantes à corps étrangers. Les diagnostiques différentiels sont les kystes épidermiques et les onchocercomes. Le traitement est chirurgical. Le mécanisme physiopathologique proposé consiste à la calcification dystrophique du muscle Dartos. Nous rapportons l'observation de Mr H.G. âgé de 45 ans sans antécédent pathologiques notables qui consultait pour de multiples lésions nodulaires kystiques du scrotum sans notion de traumatisme préalable, indolores et non infectées, évoluant depuis 10 ans. L'examen clinique du scrotum objectivait des lésions nodulaires fermes, indolores, blanchâtres mesurant de 0,3 à 1cm de diamètre. Le bilan phospho-calcique était normal. Une exérèse des nodules a été réalisée. L'examen histologique d'un nodule met en évidence des nodules kystiques calcifiés, le tissu conjonctif dermique des foyers de calcifications comportant par endroit par des foyers inflammatoires granulomateux et des follicules pileux dilatés. Il n'a pas été noté de récidive; le recul étant d'une année.

**Figure 1:**
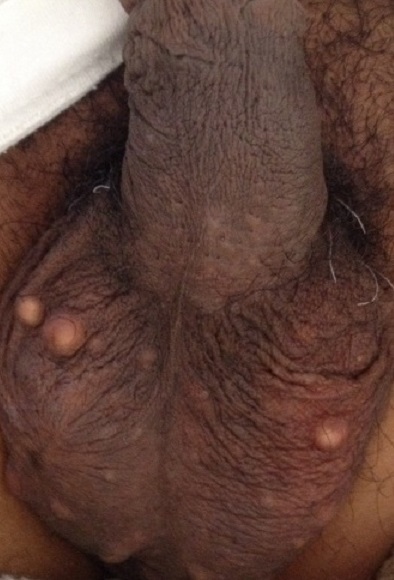
Multiples lésions nodulaires blanchâtres scrotales

